# How to improve the diet of toddlers? The feasibility of an online, habit-based intervention targeting parental feeding behaviour

**DOI:** 10.1080/21642850.2022.2134869

**Published:** 2022-10-19

**Authors:** Lisa Engels, Carlotta Mons, Enrique Mergelsberg, Emily Kothe, Kyra Hamilton, Benjamin Gardner, Gill ten Hoor, Barbara Mullan

**Affiliations:** a Department of Work & Social Psychology, Faculty of Psychology & Neurosciences, Maastricht University, Maastricht, Netherlands; b Health Psychology and Behavioural Medicine Research Group, Faculty of Health Sciences, School of Psychology, Curtin University, Perth, Australia; c Faculty of Health, School of Psychology, Deakin University, Burwood, Australia; d School of Applied Psychology, Griffith University, Nathan, Australia; e Department of Psychology, King’s College, London, UK

**Keywords:** Online intervention, healthy feeding habits, toddlers, acceptability and feasibility, behaviour change

## Abstract

**Background:**

The diet of toddlers is often not in accordance with dietary recommendations, putting them at risk of poor health outcomes later in life. Parents can struggle to provide their toddler with a healthy diet and interventions are needed. Helping parents to form healthy feeding habits may facilitate healthy feeding behaviours. Therefore, the aim of this study was to establish the feasibility of a 6-week online intervention to build healthy feeding behaviours in parents of toddlers.

**Methods:**

Parents and primary caregivers of children aged 2–3 (N = 75) were recruited to complete a 6-week online intervention consisting of 6 modules targeting habit formation, planning, goal setting, self-efficacy, interpersonal influences and picky eating. Demographics and feeding behaviours were measured with self-report at baseline and follow-up. Intervention acceptability and feasibility ratings were also gathered post-intervention.

**Results:**

Most participants were recruited online, highly educated and in a relationship. After 6-weeks, 17 participants completed the follow-up questionnaire, 11 of whom indicated that they had completed the whole intervention. Improvements were found for some feeding behaviours. Generally, participants who completed the programme reported that they found it acceptable.

**Conclusions:**

This study provides insights for future intervention development. Firstly, an online healthy feeding intervention seems to be acceptable but may need to focus on fewer change techniques. Further, engagement should be improved by including target group members and compulsory reminders. The target behaviours seem to be relevant. Online recruitment seems to be preferable and reaches parents and primary caregivers nationwide.

A balanced diet is an integral part of child health as it provides essential nutrients for growth and development while helping to prevent overweight and obesity. Childhood overweight and obesity are significant risk factors for poor health outcomes including a higher risk of type 2 diabetes, psychological disorders, and cardiovascular diseases in later life (Lifshitz, 2008). Additionally, children with obesity are five times more likely to have obesity in adulthood than other children (Simmonds et al., 2016). This suggests that ensuring a healthy diet in childhood may be protective of health risks in adulthood. Many children in Western society, however, do not meet recommendations for food and beverage consumption (Demmer et al., 2018; Mihrshahi et al., 2019) and obesity rates among children in Australia have increased over the past two decades (Australian Institute of Health and Welfare, 2017).

Given that obesity is a serious health risk factor, and that recommendations for achieving and maintaining a healthy diet are rarely met for young children, interventions targeting the prevention of obesity and overweight in young children by promoting a healthy diet are needed. During the first years of life, children learn much about food and eating (Savage et al., 2007). These early experiences with food influence later food preferences (Savage et al., 2007) which supports the need for establishing healthy eating habits from a young age. Findings also indicate that dietary interventions to prevent childhood obesity that are initiated earlier in life tend to be more successful than those initiated later in life (Birch & Ventura, 2009).

However, children’s eating behaviours are typically influenced and controlled by their parents (Birch & Ventura, 2009; Chesnut et al., 2018). Parents usually select the type and quantity of food provided to their children and determine the context in which food is eaten (Birch & Ventura, 2009). Additionally, a recent meta-analysis suggests that interventions focusing on parental feeding behaviour can be useful for changing children’s diet (Redsell et al., 2016). Therefore, designing interventions that focus on promoting healthy feeding behaviour in parents and primary caregivers may allow for earlier intervention and more consistency in children’s dietary behaviours as facilitated by their caregivers.

Despite the role that parents play in the dietary behaviours of children, comparatively few studies have explored the effectiveness of home-based, diet-only interventions (Wang et al., 2015). Therefore, little is known about the feasibility of those kinds of interventions. Given that developing and implementing behaviour change interventions involves significant financial and time commitments it is important to establish the feasibility and acceptability of any intervention prior to further dissemination (Bartholomew et al., 2001). Considering this, more research is needed that explores the feasibility of interventions that attempt to foster healthy eating among young children by promoting the healthy feeding behaviours of parents and primary caregivers.

## Theory of planned behaviour

Interventions to change behaviour must identify and then address the determinants of behaviour (Kok et al., 2017). To adequately explore the determinants of behaviour it is important to consider an appropriate theoretical framework, for example, the theory of planned behaviour (Ajzen, 1991) which has previously been used to investigate parental feeding behaviours (McKee et al., 2019). The theory of planned behaviour suggests that an individual’s attitudes (overall feeling towards enacting the behaviour), subjective norm (beliefs about the extent to which others engage in the behaviour and other people’s opinions) and perceived behavioural control (perception of control over the behaviour) predict intention to engage in a behaviour (Ajzen, 1991). An individual’s intention to engage in a behaviour, in turn, is proposed to predict actual behaviour. In addition, a direct association between perceived behavioural control and actual behaviour is proposed such that an individual’s perception of their own level of control over the behaviour is said to directly influence their performance of that behaviour.

However, although many behaviours are planned, it is understood that even if people have the intention to act in a certain way they may still fail to do so (Sheeran & Webb, 2016). Therefore, other factors aside from intention alone are expected to influence behaviour.

## Habit

Habitual behaviour is an important aspect of behaviour that is commonly studied in relation to the theory of planned behaviour (Gardner et al., 2014; McKee et al., 2019; Mullan & Novoradovskaya, 2018). Habitual behaviours form through frequent repetition in a stable setting (Lally et al., 2010) and once a habit has been formed it is performed without thinking (de Bruijn & van den Putte, 2009; Gardner et al., 2011; van’t Riet et al., 2011). This makes habits less likely to be affected by different intentions, as intentions are reasoned while habits are performed automatically (de Bruijn & van den Putte, 2009). Therefore, people often act on their habits even if they do not intend to (Gardner et al., 2020).

Considering this, future interventions targeting healthy feeding behaviours should potentially consider the impact of habit on parents’ feeding behaviours. In fact, a recent study that used an extended theory of planned behaviour that incorporated habit to investigate parental feeding behaviour found that, in combination, intention, perceived behavioural control and habit strength explained 47% of the variance in parent’s healthy feeding behaviour (McKee et al., 2019). In comparison, another recent study that examined parental feeding behaviours using the theory of planned behaviour alone, found that although attitude, perceived norms and behavioural control predicted 58.3% of the variance in parental feeding intentions, intention only predicted 25.2% of the variance in children’s actual fruit and vegetable consumption (Yee et al., 2019).

Although only a few intervention studies have focused on the role of habit for behaviour change, there are some habit-based interventions that have had promising results for habit formation (Mullan & Novoradovskaya, 2018). For example, a systematic review of habit-based weight loss interventions found that, compared to controls, the interventions that targeted habit formation resulted in significantly better weight loss outcomes for participants compared to control groups (Cleo et al., 2020).

A study by McGowan et al. (2013) provides further support for the effectiveness of a habit-based and parent-focused intervention to promote healthy feeding behaviours for children. This intervention was delivered to parents of children aged 2–6 years in the United Kingdom and addressed three parental feeding behaviours (providing fruit and vegetables, healthy snacks or healthy drinks). Habit strength, including automaticity, for all three feeding behaviours was significantly higher for parents in the intervention group than for parents in the control group. Furthermore, intake of vegetables, healthy snacks, and water was significantly greater for the intervention group than for the control group. Despite this success, the intervention was delivered face-to-face making it costly and reducing the likelihood of up-scaling (Andersson & Titov, 2014). In comparison, an online intervention offers a valuable tool to increase cost-effectiveness and accessibility (Andersson & Titov, 2014) while being generally as effective as face-to-face interventions (Vandelanotte et al., 2016).

## Barriers and enablers of healthy feeding behaviour

Given the proposed unique relationship between perceived behavioural control and actual behaviour (Ajzen, 2002) it is expected that the level of control parents feel they have over healthy feeding practices is an important element of the translation of intentions to behaviour. That is, it would be expected that if a parent has a high perception of control over their ability to feed their child a healthier diet, they will be more likely to form the intention to do so and will ultimately be more likely to engage in the behaviour successfully.

The theory of planned behaviour proposes that perceptions of behavioural control are influenced by one’s beliefs about the barriers and enablers underlying a particular behaviour (Ajzen, 1991). For example, previous findings have shown that picky eating, the consumption of an insufficient amount or a limited variety of food and/or an unwillingness to try new food items (food neophobia) (Boquin et al., 2014; Hafstad et al., 2013), is quite common in children aged 1.5–4 years and tends to act as a barrier to healthy feeding practices (Goh & Jacob, 2012; Mascola et al., 2010). If a parent perceives their child’s picky eating to be a barrier to their healthy feeding practices, they may have lower perceived behavioural control as a result and be less likely to perform the healthy feeding behaviour.

A closely related concept to perceived behavioural control is self-efficacy. Whereas perceived behavioural control is directly related to control over behaviour, self-efficacy is more appropriately described as someone’s belief about their ability to perform a behaviour (Ajzen, 1991). The two concepts are interconnected such that strengthening self-efficacy regarding a behaviour then increases an individual’s perceived level of behavioural control over that behaviour (Ajzen, 2002). In addition to the relationship between perceived behavioural control and self-efficacy, the level at which someone feels they are receiving social support has also been shown to influence their self-efficacy (Guan & So, 2016) such that a lack of social support can negatively affect self-efficacy.

Furthermore, communication skills and problem-solving skills are strongly related to self-efficacy (Erozkan, 2013). Therefore, improving an individual’s communication skills and problem-solving skills may increase their ability to deal with social conflict through building their self-efficacy. Assertiveness is a communication skill that has been shown to effectively reduce conflict and foster positive relationships (DeVito, 2013). Therefore, by improving parents’ communication skills, their self- efficacy and perceived behavioural control may increase.

## Role of time management and planning in feeding behaviour interventions

In addition to habit strength and perceived behavioural control, previous research has identified time management and planning skills as additional factors that may influence behaviour and contribute to strengthening the relationship between intentions and behaviour (Mullan & Novoradovskaya, 2018; Sniehotta et al., 2005). In a recent study, parents reported that they found planning to be essential in ensuring a healthy diet for their children (Mullan et al., 2018). However, although studies have repeatedly shown that planning influences goal achievement (Adriaanse & Verhoeven, 2018; Gollwitzer, 1999; Gollwitzer & Sheeran, 2006) parents have also stated that they frequently struggle with planning and with sticking to their plans, which hinders their ability to perform healthy feeding behaviours (Mullan et al., 2018). In line with this, it has been suggested that simply having strong intentions may not be enough to achieve one's goals (Adriaanse & Verhoeven, 2018).

The actual approach to planning is important to consider, for example, coming up with specific action plans formulated in an ‘if-then’ structure, has been found to facilitate goal achievement and to increase the automaticity of goal-directed responses (Gollwitzer & Sheeran, 2006; Hagger et al., 2016). Other forms of planning that have also been found to facilitate goal achievement are coping planning (Pakpour & Sniehotta, 2012; Sniehotta et al., 2005) and action planning (Adriaanse & Verhoeven, 2018). Coping planning involves anticipating potential barriers and forming plans on how to overcome them while action planning means the preplanning of certain behaviours. Recent research found that while intention was a strong predictor of dietary behaviours the relationship was moderated and mediated by both action and coping planning respectively (Hamilton et al., 2017). These findings suggest that both planning and goal setting are important factors to consider when developing a behaviour change intervention that aims to help parents ensure a healthy diet for their children.

## The current study

The aim of this study was to assess the feasibility and acceptability of an online parental feeding intervention targeting three healthy feeding behaviours which prior research identified as ones which parents often struggle to successfully engage in (Mullan, 2018). Namely, providing a healthy breakfast, providing healthy drinks (i.e. water and milk), and including vegetables at dinner. This was done to increase the relevancy of the programme and to allow for the opportunity to explore whether the intervention was more or less successful for different behaviours. However, the programme was not specifically designed for particular behaviours but rather was targeted towards parental feeding behaviour in general.

Assessing the feasibility and acceptability of the intervention were the primary objectives. Secondary objectives were to document the characteristics of participants taking part in this healthy feeding intervention and to explore the potential impact of the intervention on parental feeding behaviours. It was expected that the intervention would be acceptable to participants and that participants’ feeding behaviours would become healthier thereby improving their toddlers’ diet.

## Methods

### Study design and procedure

This paper includes a description of the structure and content of an intervention targeting parental feeding behaviours as well as a quantitative analysis of the feasibility and acceptability of the intervention. Data were obtained by a pre–post design. The programme was advertised in two researcher interviews with local radio stations and advertisements on social media networks, supermarket notice boards, and childcare centres. Upon completion of the modules and the follow-up measures, participants could claim an AUD 30 shopping voucher as compensation for their time and effort. After providing their consent, participants completed a baseline questionnaire and received the link to the online intervention. Subsequently, participants took part in the six-week *Creating Healthy Eating Environments for Toddlers by Adopting Habits* (CHEETAH) online intervention by following the instructions and completing the modules provided on the intervention’s website.

Each week participants were asked to complete a one-hour module. Participants chose when to do the module and were able to split the modules into subparts as needed. Participants were able to choose to receive reminders which were sent out via e-mails once a week prompting participants to continue through the programme. Upon completion of the 6-week intervention period, participants were asked to fill in a follow-up questionnaire which duplicated the baseline questionnaire and included additional questions about the acceptability of the intervention. Ethical approval was granted by the University’s Human Research Ethics Committee (HRE2019-0094).

### Participants

Eligible participants were a parent or primary caregiver of a child aged two to three. Parents and primary caregivers were required to be able to speak and understand English. Participants were ineligible if their child had a chronic disease affecting their ability to eat a typical/unrestricted diet. Considering the primary objective of this study to assess the feasibility and acceptability of the intervention prior to a larger trial assessing behaviour change, we aimed to recruit a minimum of 12 participants (Moore et al., 2011). However, as the intervention was previously untested we anticipated a large drop-out, especially given the extended time frame and self-directed aspect of the intervention. Therefore, we endeavoured to over-sample with the aim to retain at least 12 participants. As almost all of the first 30 participants came from the same Facebook group an ethics amendment was submitted that would allow for the recruitment of additional participants through childcare centres and supermarket notice boards. A total of 75 participants gave consent to participate in the study, completed the baseline survey and were provided with a link to the intervention materials. Findings from this feasibility study will be used to inform sample size calculations for any subsequent trials.

### Intervention

The intervention consisted of six weekly modules, with interactive and reflective tasks to build healthy feeding behaviours. Throughout the programme, parents worked on a personalised goal related to one of three behaviours they were asked to select (see Module 1). Each module focused on one intervention component to help parents and primary caregivers of toddlers reach their goal. Given findings from the literature and content of other interventions this programme focussed on habit formation, planning and goal setting, self-efficacy, social influences and support, and habit strengthening.

Each module included self-reflection tasks whereby participants were provided with examples and text boxes to serve as prompts and then instructed to think of a particular situation and to write down their thoughts and reflections. Participants were encouraged to contact the research team informally to provide them with any feedback.

#### Content of the six modules

##### Module 1

The first module focussed on providing a foundation for habit formation that would be built on throughout the remaining modules. Participants first received general information about what habits are, how they develop, and how they may help with ensuring their child engages in healthy eating behaviour. Next, participants were asked to identify the context in which they usually performed the three feeding behaviours. After that, participants were asked to choose one of the three behaviours to work on throughout the programme and to describe how they wished to change the behaviour in concrete terms.

##### Module 2

This module was centred on action planning and goal setting to help parents build healthy routines and improve their time management skills. Participants learnt what makes a good goal and were introduced to the concept of SMART goals (Locke & Latham, 1990, 2002). Participants were then asked to re-frame their chosen healthy feeding behaviour as a goal, thus building on the content of the prior module. Participants were also encouraged to use a meal planner and shopping list template throughout the duration of the programme. Lastly, participants were encouraged to reflect on the goal they set in Module 1 and to revise/refine it as needed.

##### Module 3

In the third module, participants learnt about self-efficacy and barriers. Specifically, participants learnt how to overcome barriers, that may hamper their goal achievement, and were shown how to use coping planning. They were also guided through the formation of implementation intentions to facilitate goal achievement. Finally, participants were asked to take another look at their goal and revise it if necessary.

##### Module 4

The fourth module was concerned with interpersonal influences. Specifically, participants learnt how to manage conflict with other adults such as partners, family members, and friends. Participants were asked to think about one person who supported their healthy feeding goal and one person that they wanted more support from. Parents were provided with information on strategies suggested by the Australian government to address conflict and to increase assertiveness, such as anger management, improving communication and teamwork, and disclosing one’s needs (Better Health Channel, 2022).

##### Module 5

This module was about helping to manage disagreement with one’s own child. Participants received tips that could help them take control and to prevent or reduce conflict with their child (Henry & Borzekowski, 2011). They were introduced to some techniques that could improve picky eating, such as repeated exposure to disliked food (Cooke, 2007; Hetherington et al., 2015; Wardle et al., 2003), and were asked to pick one of them and specify how they would action it.

##### Module 6

The last module of the programme provided advice on how to strengthen any newly formed behaviours and how to effectively merge them into one’s daily routine moving forwards. Participants were introduced to setting graded tasks (i.e. starting with easy tasks and increase the difficulty until goal behaviour is reached; Kelder et al., 2015) and provided with a goal template to use in the future.

## Data collection

### Baseline measures

#### Demographics

Participants provided details on their age, their child’s age, the number of children they had, their educational background, employment status and relationship status.

#### Parental body mass index (BMI)

Participants provided their height and weight from which parental BMI was calculated. Previous research has found it to be associated with child’s health behaviours and weight (Lee et al., 2019).

#### Index of relative socio-economic advantage and disadvantage (IRSAD)

To determine which decile of the IRSAD participants fell into they were asked to provide their postcodes. The IRSAD provides information about the economic and social conditions of people living within certain areas (Australian Bureau of Statistics, 2018). IRSAD deciles range from 1 to 10 with higher values indicating a relative lack of disadvantage and greater advantage (Australian Bureau of Statistics, 2018).

#### Feeding behaviours

To measure feeding behaviours participants were asked to indicate, using a timeline follow-back method (Sobell & Sobell, 1992), how many servings of drinks and vegetables their child had and what their child had eaten for breakfast for a period of three days. Participants were asked to pick three recent days, with at least one day on the weekend to account for differences depending on the day of the week, and were asked to indicate whether any special events took place on the days. The healthiness of the items provided was rated on a 5-point Likert scale based on the Australian Health Star Rating (Commonwealth of Australia, 2019). For the healthiness of breakfast, the health star rating for each item was added and divided by the total number of items, mean scores were calculated with higher scores indicating a healthier breakfast. Mean scores for the provision of healthy drinks and servings of vegetables were also calculated.

### Follow-up measures

#### Primary outcomes: engagement, adherence and acceptability

Acceptability and feasibility questions were based on previous studies examining the acceptability and feasibility of food-related online interventions (Kothe & Mullan, 2012; Sainsbury et al., 2015).

To determine the level of engagement they had with the programme content participants were asked which modules they had completed and how much time they spent using the website. Participants were categorised as having completed the programme when they indicated that they completed at least 5 out of the 6 modules of the intervention.

Adherence was operationalised as the amount of time that participants reported having spent attempting to apply what they learnt using the programme.

Measures of acceptability of the intervention included attrition and ratings of programme content. Attrition was calculated as the percentage of individuals who provided consent to take part but did not complete the final survey. Participants rated, using sliding scales from 1 to 100 (1 =  ‘Not at all’, 100 = ‘Very’), whether the programme content was: annoying; interesting; credible; logical; easy to understand; personally relevant; confusing; comprehensive; too long and/or useful.

#### Secondary outcomes: parental

To measure feeding behaviours post-intervention participants were asked to fill in the questionnaire using the timeline follow-back method (described above) again. Any changes in parental feeding behaviours were used to assess the likelihood of harm resulting from participation in the programme.

## Analysis

Initially, it was planned to use dependent samples t-tests for differences between those who participated in the follow-up questionnaire and either completed 5 or more modules of the programme (completers) or completed less than 5 modules of the programme (non-completers) related to acceptability and feasibility ratings and independent samples t-tests for investigating participants’ behaviour scores pre-and post-intervention. However, given the small sample size due to dropout, this analysis was not possible. Instead, the outcomes were assessed as described below.

### Primary outcomes: engagement, adherence and acceptability

Two primary outcome variables were compared between completers and non-completers. Engagement level, defined as time spent on the website and adherence to the intervention, in the form of time spent applying what was learned.

Acceptability of the intervention was assessed comparative to the attrition rate reported by Sainsbury et al. (2013) in their 6-week, online, TPB dietary behaviour intervention. Namely, an attrition rate of ≤50% would be interpreted as indicating that the intervention was acceptable to participants. Additionally, ratings of intervention content were categorised as endorsing or refuting the characteristic if the median rating was above 70 or below 30 respectively.

### Secondary outcomes: parental feeding behaviours

The Wilcoxon signed-rank test was used to examine the behaviour scores for breakfast, drink and vegetables pre- and post-intervention. Any differences in behaviour scores, irrespective of statistical significance, should indicate that there are no negative changes pre- and post-intervention that may indicate a risk of harm as a result of participation in the intervention.

## Results

### Participants

Seventy-five parents of children aged 2–3 gave informed consent and received a link to the programmes website. Out of the 75 participants that signed up 28 chose to receive weekly reminders to continue participation. Table 1 outlines the characteristics of the study participants at baseline and follow-up.

**Table 1.** Characteristics of participants at baseline and follow-up and those who dropped out.

Characteristic	*Baseline*		*Drop-out*		*Follow-up*	
	(*n *= 75)		(*n *= 58)		(*n = *17)	
	*M*	*SD*	*M*	*SD*	*M*	*SD*
Age	33.8	4.4	33.9	4.3	33.7	5.0
BMI	27.6	6.7	27.5	6.2	26.2	6.0
IRSAD decile	7.0	2.6	7.2	2.5	6.5	2.9
Children’s age	2.7	1.1	2.7	1.0	2.8	1.3
In a relationship						
Yes	*n *= 73 (97.3%)		*n *= 55 (94.8%)		*n *= 16 (94.1%)	
Number of children						
1	*n* = 33 (44.0%)		*n* = 28 (48.3%)		*n *= 6 (35.3%)	
2	*n* = 32 (42.7%)		*n* = 23 (39.7%)		*n *= 8 (47.1%)	
3+	*n = *10 (13.3%)		*n = *7 (12.0%)		*n *= 3 (17.6%)	
Employment						
Part-time	*n = *23 (30.7%)		*n = *16 (27.6%)		*n *=* *7 (41.2%)	
Full-time	*n = *17 (22.7%)		*n = *15 (25.9%)		*n *= 2 (11.8%)	
Full-time parent or carer	*n = *20 (26.7%)		*n = *14 (24.1%)		*n = *6 (35.3%)	
Other	*n =* 15 (19.9%)		*n =* 13 (22.4%)		*n *= 2 (11.8%)	
Education						
High school equivalent	*n* = 6 (8.0%)		*n* = 4 (6.9%)		*n *= 2 (11.8%)	
TAFE* certificate	*n *= 22 (29.3%)		*n *= 17 (29.3%)		*n* = 5 (29.4%)	
Undergraduate degree	*n* = 26 (34.7%)		*n* = 19 (32.8%)		*n *= 7 (41.2%)	
Postgraduate degree	*n* = 21 (28.0%)		*n* = 18 (31.0%)		*n *= 3 (17.6%)	
State of residence						
Western Australia	*n* = 47 (62.7%)		*n* = 37 (63.8%)		*n *= 11 (64.7%)	
Other Australian State	*n* = 28 (37.3%)		*n* = 21 (36.2%)		*n *= 6 (35.3%)	
Remoteness of location						
Metropolitan	*n* = 55 (73.3%)		*n* = 44 (75.9%)		*n *= 11 (64.7%)	
Rural	*n* = 20 (26.7%)		*n* = 14 (24.1%)		*n *= 6 (35.3%)	
Advertisement						
Online	*n* = 62 (82.7%)		N/A		N/A	

Note: *M *= Mean, *SD *= Standard deviation, *TAFE: Technical and Further Education. Public vocational education and training in Australia (Wheelahan et al., 2018).

### Primary outcomes: engagement, adherence and acceptability

All participants who completed the baseline questionnaire received an invitation to complete the follow-up questionnaire regardless of whether they had completed the intervention or not. Fifteen out of 75 participants at baseline completed the follow-up questionnaire (20.0%), however an additional 2 participants started the questionnaire but did not complete the outcome measures and have not been included in reporting of the reporting of the primary outcomes. Of the 15 who completed the follow-up questionnaire, 11 indicated they had completed at least 5 out of 6 modules and were therefore categorised as ‘Completers’ of the programme while the remaining 4 who completed less than 5 modules were catagorised as non-completers of the programme. Thus, the resulting attrition rate from the study was 85%.

Table 2 provides an overview of the scores for each acceptability measure, as well as engagement and adherence. The medians of completers and non-completers indicate that the content was rated as interesting, credible, logical, easy to understand, relevant, comprehensive, and useful. Conversely, the content was rated as neither annoying nor confusing. Differences between completers and non-completers indicate that non-completers more often rated the intervention as being ‘too long’ compared to completers.

**Table 2.** Engagement, adherence and acceptability ratings.

Evaluation variable	*Completers* (*n *= 11)			*Non-completers* (*n *= 4)		
	*Mdn*	*Min*	*Max*	*Mdn*	*Min*	*Max*
Time (minutes) spent on the website	17.0	9	60	26.5	14	95
Time (minutes) spent practising	51.0	1	120	19.5	9	40
Annoying*	7.0	0	18	17.5	2	60
Interesting*	86.0	50	100	67.5	21	93
Credible*	80.0	47	100	77.5	70	85
Logical*	82.0	54	100	63.0	43	81
Easy to understand*	81.0	46	100	84.0	35	98
Relevant*	85.0	51	100	73.0	48	89
Confusing*	3.0	0	39	26.0	1	71
Comprehensive*	80.0	34	100	66.0	7	80
Too long*	7.0	0	80	84.0	33	94
Useful*	87.0	52	100	77.0	53	98

Note: *Mdn* = Median.

*1 =  ‘Not at all’, 100 = ‘Very’.

Trends indicate that completers rated the content as less annoying, more interesting, credible, logical, easier to understand, more relevant, less confusing, more comprehensive, and more useful than non-completers.

In regard to engagement and adherence, completers reported that they spent less time on the website than non-completers but more time attempting to apply the knowledge they gained from the programme.

### Secondary outcomes: parental feeding behaviours

Due to the small sample size there was insufficient statistical power to adequately assess the impact of the intervention on parental feeding behaviours. Instead, changes in parental feeding behaviours were used to assess whether or not participation in the programme was likely to result in harm.

Table 3 outlines the behaviour scores pre- and post-intervention for the 11 participants who completed the intervention. Wilcoxon Signed-Rank Tests indicated that participants had significantly improved scores for serving vegetables on the Timeline follow-back (z = −2.251, *p* < .024) with a large effect size (r = .48). The scores for drinks and breakfast on the Timeline follow-back did not change significantly (z = −1.293, *p* < .196; z = −.561, *p* < .575). Effect sizes were medium for drinks (r = .28) and small for breakfast (r = .12). As improvements or no significant changes were detected, participation in the programme is considered to be unlikely to cause significant harm in regard to the target behaviours.

**Table 3.** Behaviour scores for completers (*n* = 11).

Behaviour	*z*	*p*	*r*	*Mdpost*	*Mdpre*
Vegetables	−2.25	.024	.68	1	2
Drinks	−1.29	.196	.39	4	5
Breakfast	−0.56	.575	.16	4	4.16

Note: *z:* Z-value*, p: p*-value*, r:* effect size*, Mdpre:* Median pre-intervention*, Mdpost:* Median post-intervention.

## Discussion

The objective of this study was to assess the feasibility and acceptability of an intervention to promote healthy feeding behaviours in parents and primary caregivers of toddlers. The findings provided insights into how to further develop and adjust the intervention to improve fit for future participants and increase engagement.

### Primary outcomes: engagement, adherence and acceptability

Participants who completed the follow-up questionnaire and completed the intervention found the intervention was acceptable and feasible. The majority of the participants, however, did not complete the intervention or the follow-up questionnaire. The dropout rate was high with parents either dropping out immediately or after finishing the first two to three modules. Despite this being a common problem with online interventions, which often have dropout rates as high as 84% (Reinwand et al., 2015), the high drop-out rate for this study suggests that changes to the intervention are needed to make it more feasible and acceptable to a larger cohort.

A possible explanation for the high drop-out rate could be that non-completers rated the programme as being too long. In line with this, most dropout was after 2–3 weeks. This finding is supported by post-survey data of non-completers indicating that they found the intervention too repetitive and long. Overall, this is problematic as 2–3 weeks is not enough time to form habits. For habits to form, participants must engage regularly and sufficiently long with the intervention content and practice the behaviour in their daily routine (Gardner et al., 2011). Additionally, the intervention may have been too broadly focused and may have benefitted from exploring fewer techniques to facilitate behaviour change. In future research, participants could be randomised to different conditions and encouraged to utilise one specific technique with results compared between groups. This may reduce participant burden and heighten engagement.

Another reason for the high dropout might be related to participants forgetting to engage with the intervention. Although this study included weekly reminders, receiving them was optional and most participants did not sign up to receive them (N = 47). Due to the modular nature of the intervention participants were required to engage weekly and without reminders, they may have forgotten to keep up the intervention. This is in line with prior research suggesting that receiving reminders is important for engagement with online interventions (Milward et al., 2018).

### Secondary outcomes: parental feeding behaviour and participants’ characteristics

Although participants were directed to choose a behaviour to focus on throughout the modules, they still received information regarding all three behaviours therefore there was the potential for them to change their behaviour in regard to all three. The findings suggest that improvements were made in serving vegetables at dinner while serving healthy drinks and providing a healthy breakfast did not show any relevant changes. This may be because participants might have chosen to work on serving vegetables, instead of the other two target behaviours. In addition, the behavioural measure showed that the healthiness of drinks and breakfast provided by the participants was already very high, which could explain the lack of improvement (i.e. ceiling effect). To determine whether choice of behaviour impacted the efficacy of the intervention future research should ensure the participants choice of behaviour was recorded.

The characteristics of the participants indicate that the intervention was most interesting for parents/primary caregivers in their thirties (*M *= 33.8, *SD* 4.4) in a relationship (97.5%) with 1–2 children (86.7%) living in urban areas (73.3%). Furthermore, most participants had at least an undergraduate degree or TAFE certificate (92.0%), indicating that most participants had a high education level. Additionally, the average BMI among participants was 27.6, which implies that participants, on average, were overweight. However, calculation of parental BMI relied on participant’s providing estimates of their height and current weight which are both commonly inaccurate resulting in lower BMIs (Gorber et al., 2007). Participants’ characteristics also showed that most participants were recruited through online advertisements. Moreover, the programme was able to reach participants nationwide, with 37% from a state other than Western Australia despite the programme being mainly advertised in Western Australia.

### Strengths, limitations and future directions

A strength of this study was to clearly document the intervention and associated content. To adequately determine the effectiveness of an intervention it is necessary to clearly identify the behavioural determinants that the intervention is attempting to affect change upon. By clearly outlining the design and aims of this research we were able to be transparent about how we hoped to create change. Additionally, by conducting a comprehensive feasibility analysis we have gathered extensive evidence for the acceptability of this type of intervention. We have also provided clear directions for any further development or adaption of the intervention below.

The learnings from this study include some potential changes to the intervention that may help to increase engagement. Although previous research by Mullan (2018) was used to inform the choice of target behaviours, there was limited further consultation with the target population for this research. To tailor the programme as best as possible to participants’ needs, more members of the target group should be involved in the future. Having more target group members involved might also allow for the detection of potential issues or improvements in the development stage (Eldredge et al., 2016).

An additional limitation that should be considered is that information about completion of the modules and time spent on the website was only available via self-report for those who completed the follow-up survey. Instead, the website could be designed to collect data on access to the website and time spent on each section to gain further insight into module completion and engagement. Additionally, drop-out could be pinpointed to specific modules and this could provide further direction for improvement. It is necessary to provide this kind of detail to allow for feasibility and acceptability to be tracked throughout different aspects of online interventions and to allow for comparison between studies. Although it was decided to conduct the intervention online to ensure the cost was as low as possible, we did not conduct an analysis of the costs of the intervention. Future studies should record this data to ensure that interventions are not only effective but also economically viable.

Additionally, as this was a feasibility study the number of participants was lower that would be needed to provide an in-depth comparison between those who completed and those who did not complete the intervention. Lastly, prior research (Milward et al., 2018) suggests that reminders may be important to keep participants involved in the intervention. Unlike the current study, receiving reminders should be compulsory in future interventions to increase engagement with the intervention and to lower the dropout rate.

### Conclusion

Overall, this study established that an intervention aimed at promoting healthy feeding behaviours in parents and primary caregivers of toddlers that was acceptable for the participants who completed the intervention. However, acceptability and feasibility overall were limited by the high drop-out rates. Thus, it is not possible to conclude that the intervention is feasible in its current format. The main focus of future intervention development should be to increase engagement with online interventions. As supported by our findings, potential avenues to increase engagement may include having a group of potential participants involved in the intervention development (Eldredge et al., 2016) and including compulsory reminders (Milward et al., 2018). Furthermore, targeting fewer change techniques may additionally increase feasibility. The three target behaviours, especially serving vegetables, seem to be relevant and improvable. Lastly, future online intervention should focus on advertising online as it seems to be more successful than traditional methods and reaches potential participants nationwide. Despite the limited success of the intervention, this study provides important insights that cannot only be used to further develop and improve the current.
